# Non‐*albicans Candida* in Vulvovaginal Candidiasis: Antifungal Resistance and Expression of *ERG11*, *CDR1*, *CDR2*, and *MDR1* Genes

**DOI:** 10.1002/mbo3.70123

**Published:** 2025-11-30

**Authors:** Fatemeh Zahra Ranjbar Golafshani, Firoozeh Kermani, Soheila Abbaszadeh Godarzi, Saeid Mahdavi Omran

**Affiliations:** ^1^ Parasitology and Medical Mycology Department, Faculty of Medicine Babol University of Medical Sciences Babol Iran; ^2^ Infectious Diseases and Tropical Medicine Research Center, Health Research Institute Babol University of Medical Sciences Babol Iran; ^3^ Student Research Committee Babol University of Medical Sciences Babol Iran; ^4^ Obstetrics and Gynecology Department, Faculty of Medicine Babol University of Medical Sciences Babol Iran; ^5^ Infertility and Reproductive Health Research Center, Health Research Institute, Rouhani Hospital Babol University of Medical Sciences Babol Iran

**Keywords:** gene expersion, *Nakaseomyces glabratus*, *Pichia kudriavzevii*, vulvovaginal candidiasis

## Abstract

The rise in azole resistance among *Nakaseomyces glabratus* and *Pichia kudriavzevii* in recurrent vulvovaginal candidiasis presents a growing public health challenge. This study investigated the expression of antifungal resistance‐related genes (*ERG11*, *CDR1*, *CDR2*, and *MDR1*) in clinical resistant (CR) and clinical and laboratory resistant (CLR) strains of these yeasts. Cervicovaginal samples from patients with recurrent infections were collected, microscopically examined, and cultured. Yeast species were identified phenotypically and genotypically, followed by drug sensitivity testing. Total RNA was extracted, reverse transcribed to complementary DNA, and real‐time polymerase chain reaction was used to quantify target gene expression, comparing results to drug‐sensitive controls. Non‐*Candida albicans* species constituted 29% (45 cases) of the isolates, with *N. glabratus* (68%) and *P. kudriavzevii* (17%) being the dominant species. Other species included *Candida parapsilosis, Meyerozyma guilliermondii*, *Candida orthopsilosis*, *Saccharomyces cerevisiae*, and *Rhodotorula mucilaginosa*. Coinfections with *P. kudriavzevii/C. albicans* and *N. glabratus/C. albicans* were also observed. Ketoconazole, itraconazole, and 5‐flucytosine demonstrated the best antifungal activity against most species. However, some *N. glabratus* isolates were resistant to miconazole, clotrimazole, and amphotericin B, while all *P. kudriavzevii* isolates resisted clotrimazole. Overexpression of the *CDR1* gene was noted in *N. glabratus* (CR, 21.53 ± 1.26; CLR, 84.96 ± 0.67), and the *ERG11* and *CDR1* genes in *P. kudriavzevii* (*ERG11* for CR, 28.56 ± 2.16; *CDR1* for CLR, 35.89 ± 0.35). These results indicate that even in cases where an isolate is classified as susceptible by drug susceptibility testing, elevated gene expression may persist, and treatment should not be discontinued.

## Graphical abstract

The rise in azole resistance among *Nakaseomyces glabratus* and *Pichia kudriavzevii* presents a growing public health challenge. These results indicate that even in cases where an isolate is classified as susceptible by drug susceptibility testing, elevated gene expression may persist, and treatment should not be discontinued.

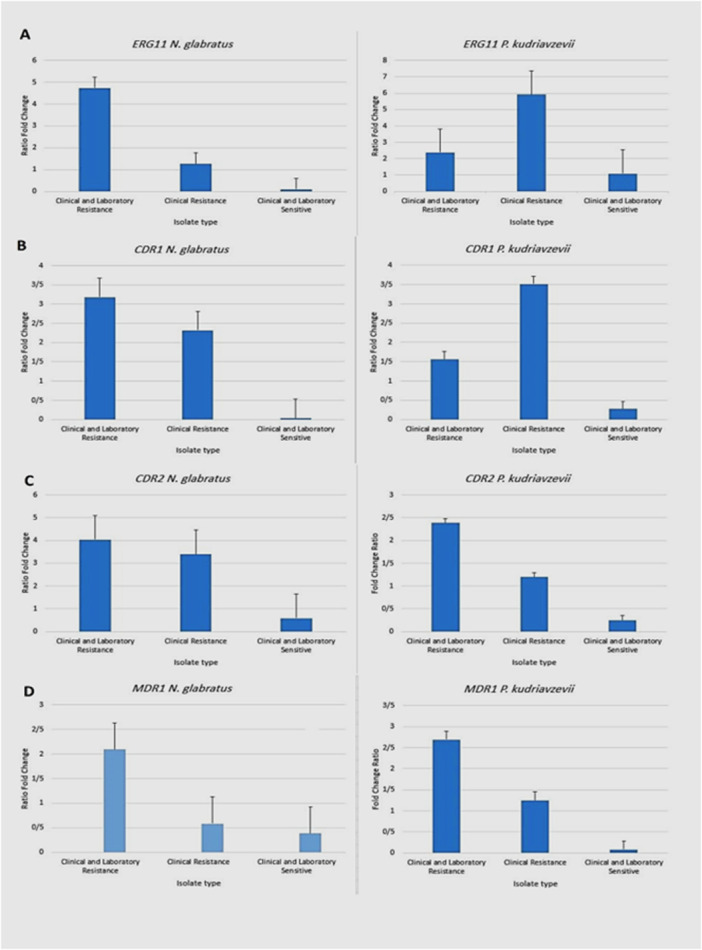

**Abbreviations**
CDR1: *Candida* drug resistance1 gene
CDR2: *Candida* drug resistance2 gene
CLR: clinical and laboratory resistant
CLS: clinical and laboratory sensitive
CR: clinical resistant
ERG11: ergosterol 11 (sterol 14‐demethylase) gene
MDR1: multidrug resistance1 gene
PMA1: plasma membrane ATPase 1 gene

## 1 Introduction

Vulvovaginal yeast infections are common among women and are mainly caused by various yeast species, including *Candida*. Recently, there has been a rise in diseases caused by non‐*albicans Candida* (NAC) species (Jannati et al. 2024; Fernandes et al. 2023). These species are linked to recurrent infections and exhibit significant resistance to antifungal agents. Azoles, a well‐known and widely used group of antifungal agents, have been effective and well tolerated in treating this type of infection (Makanjuola et al. 2018). However, species such as *Nakaseomyces glabratus* and *Pichia kudriavzevii* demonstrate intrinsic resistance to fluconazole, and the resistance of these species to azoles poses a significant threat to human health. Exposing fungi to antifungal agents creates strong selective pressure, leading to the development of drug‐resistant mutations and the emergence of microbial resistance to drugs (Schikora‐Tamarit and Gabaldón 2024; Daneshnia et al. 2023). Consequently, antifungal drug resistance has become a pressing concern in clinical settings (Czajka et al. 2023).

Multiple mechanisms contribute to azole resistance in fungi. These mechanisms include mutations in genes involved in ergosterol biosynthesis, which provide tolerance to the toxic intermediates that accumulate when azoles inhibit sterol 14‐α‐demethylase (Lee et al. 2023). Other mechanisms include overexpression of the drug target lanosterol 14‐α‐demethylase, single or multiple point mutations in the drug target, and overexpression of drug efflux pumps (Esfahani et al. 2024). Although azole‐resistant clinical isolates of *Candida*, the predominant human fungal pathogen, may possess multiple resistance mechanisms, the most common cause of high‐level azole resistance is an energy‐dependent drug efflux pump (Sanchez Villacreses 2023). A study on resistant mechanisms to azole drugs identified changes in the target enzymes, primarily caused by overexpression and mutations of the *ERG11* gene (Maheronnaghsh et al. 2022). However, numerous studies consistently report that the most common resistance mechanism observed is the overexpression of genes encoding ABC membrane transport proteins, specifically *CDR1* and *CDR2 (*Liu et al. 2021 *)*. Furthermore, several studies have established that the upregulation of the *MDR1* gene is primarily associated with fluconazole‐resistant isolates (Esfahani et al. 2024; Sanchez Villacreses 2023; Maheronnaghsh et al. 2022; Khosravi Rad et al. 2016; Morais Vasconcelos Oliveira et al. 2021; Ranjbar Golafshani et al. 2024).

Drug resistance in clinical isolates, especially those that show resistance in clinical and laboratory conditions, is a complex and challenging issue in the field of clinical mycology (Hossain et al. 2022). This phenomenon, which is also known as acquired resistance in clinical conditions, is the problem of the emergence of drug resistance in microorganisms that are exposed to the selective pressure of drugs during hospital infections (Salam et al. 2023). This resistance is often caused by the horizontal transfer of resistance genes and random mutations in the genomes of bacteria and indicates the ability of microorganisms to adapt to specific environmental conditions and develop mechanisms of resistance to drugs (Michaelis and Grohmann 2023). However, it may also exist in fungi (Morogovsky et al. 2022). Considering the importance of this topic in the treatment of infections and increasing the duration of patients' involvement, the necessity of conducting comprehensive and detailed studies in this field is felt more and more. So far, there has been no extensive research on the direct comparison of the drug resistance of only resistant clinical isolates with that of resistant isolates (Rogers et al. 2022).

Despite considerable advancements in the identification of antifungal resistance mechanisms, a notable gap in the current body of knowledge persists. Specifically, there has been insufficient investigation into a comprehensive and direct comparison of the expression patterns of key resistance genes (*ERG11, CDR1, CDR2*, and *MDR1*) between clinical isolates of *N. glabratus* and *P. kudriavzevii*. This comparison should focus on those isolates that have developed resistance in clinical settings (clinically resistant isolates or clinical resistant [CR]) versus those that have acquired resistance under laboratory conditions (laboratory resistant isolates or clinical and laboratory resistant [CLR]). Such an analysis could yield critical insights into the phenotypic and genetic distinctions between resistance acquired in natural infection contexts and resistance induced in controlled laboratory environments. This methodological approach would facilitate the identification of key molecular pathways that are instrumental in the emergence of resistance in human infections, thereby informing the development of more targeted diagnostic and therapeutic strategies. A comprehensive understanding of resistance mechanisms in these NAC strains, as well as the interactions between host environments and drug stressors that activate these mechanisms, is essential for effectively managing azole‐resistant infections and reducing rates of treatment failure.

## 2 Materials and Methods

### 2.1 Patients and Collecting Isolates

The study was conducted by Babol University of Medical Sciences in adherence to the ethical guidelines outlined in code IR.MUBABOL.HRI.REC.1402.138. The study involved a cohort of 402 women aged 18–60 years who presented with complaints of pruritus, pain, and vaginal discharge, along with recurrent suspected infections associated with cervicovaginal candidiasis. These participants were referred to the Omid Clinic affiliated with Babol University of Medical Sciences. Demographic data, as well as clinical signs and symptoms such as erythema, discharge, pruritus, pain, and burning sensations, were systematically recorded. Recurrent cervicovaginal candidiasis (RCVC) was defined as the occurrence of more than four episodes within 1 year.

Sampling was conducted for all patients, and following the receipt of positive mycological culture results, a treatment regimen was prescribed by the gynecologist and obstetrician. This regimen included vaginal clotrimazole ointment and fluconazole capsules (150 mg) for a duration of 7 days, in conjunction with metronidazole tablets (500 mg) administered twice daily for 7 days, as well as cefixime tablets (200 mg) taken twice daily for 7 days. Additionally, all patients received counseling regarding hygiene practices and the modification of detrimental lifestyle habits.

Patients returned for a follow‐up consultation after 2 weeks; except for 11 cases, the majority of patients exhibited clinical recovery. Sampling was repeated for these 11 patients, and upon review of the culture and microscopic findings, a second treatment course was initiated, consisting of vaginal miconazole ointment for 7 days and fluconazole capsules (150 mg) for 30 days. Patients were instructed to adhere to all recommended hygiene measures.

After a subsequent 40‐day period, patients returned for evaluation of the treatment's efficacy, and significant improvement was observed in all cases, with no reports of residual symptoms. A third smear was performed to further confirm the treatment outcomes.

Furthermore, all patients underwent a clinical re‐evaluation after 6 months, at which point recovery was confirmed.

### 2.2 Exclusion Criteria

Infections resulting from *Trichomonas vaginalis*, bacterial vaginosis, vulvar skin infection, pregnancy, and prior usage of topical or systemic antifungal agents within the previous 2 weeks were deemed exclusion criteria.

### 2.3 Yeast Identification

Sampling was done by a gynecologist using a speculum to collect vaginal discharge from the posterior fornix and cervical discharge with two sterile cotton swabs, which were then placed in 3 mL of sterile saline. The vagina and cervix were checked for any abnormalities. For further examination, cervicovaginal Papanicolaou (Pap) stained smears were collected from the patients and submitted to the pathology laboratory. Potassium hydroxide (KOH 10%) solution was used in the preparation of temporary wet mounts for the direct examination of specimens for yeast or pseudomycelium forms. Other samples were inoculated aerobically onto Sabouraud Dextrose Agar (SDA) (Merck, Germany) supplemented with chloramphenicol (Sc) and incubated at 35°C for a period of 4 days. Initial growth of yeast species was observed on CHROMagar *Candida* colony medium (CHROMagar *Candida*, France). The plates were examined daily for yeast growth and colony color. Microscopic slides were prepared directly from each colony for yeast confirmation. The germ tube test was conducted in serum at 37°C for a duration of 2–3 h, and the microscopic morphology was examined on corn meal agar (HighMedia, India) supplemented with 1% Tween 80 (Merck, Germany) and incubated at 42°C–45°C for 24–48 h.

### 2.4 DNA Extraction

Genomic DNA was extracted using the glass bead disruption method (Yamada et al. 2002). Briefly, yeast cells were added to a mixture consisting of 300 μL of lysis buffer (10 mM Tris, 1 mM ethylenediaminetetraacetic acid [EDTA], pH 8, 1% sodium dodecyl sulfate, 100 mM NaCl, 2% Triton X‐100), 300 μL of phenol chloroform solution (1:1), and 300 μL of glass beads (0.5 mm in diameter). The mixture was vigorously shaken for 5 min, followed by centrifugation at 10,000 rpm for 5 min. The supernatant was carefully transferred to a new tube, and an equal volume of chloroform was added. After gentle mixing and centrifugation, the supernatant was again transferred to a new tube. To precipitate the DNA, 2.5 mL of cold absolute ethanol and 0.1 M sodium acetate, 3 M were added to the supernatant. The mixture was gently shaken and centrifuged at 10,000 rpm for 10 min at 4°C. The pellets were washed with 70% ethanol and resuspended in 100 μL of TE buffer (10 mM Tris, 1 mM EDTA). Finally, the DNA was stored at −20°C.

### 2.5 RCR‐RFLP and Sequencing Analysis

A polymerase chain reaction (PCR) reaction volume of 25 μL was prepared by combining 12.5 μL of Mastermix and 1 μL of DNA extracted from each isolate. The amplification of the internal transcribed spacer (ITS) region was performed using the primers ITS1 (5′‐TCCGTAGGTGAACCTGCGG‐3′) and ITS4 (5′‐TCCTCCGCTTATTGATATGC‐3′). To this mixture, 10.5 μL of water diethylpyrocarbonate (DEPC)‐treated was added and thoroughly mixed. The PCR reaction consisted of an initial denaturation step at 94°C for 5 min, followed by 35 cycles of secondary denaturation at 94°C for 1 min, annealing at 56°C for 1 min, and extension at 72°C for 1 min. The final cycle included a final extension step at 72°C for 7 min. The amplified PCR products were subjected to 1.5% agarose gel electrophoresis in 0.09 M Tris–borate–EDTA (TBE) buffer and stained with Safe stain. Subsequently, the PCR products were digested in a final reaction volume of 31 mL containing 18 mL of deionized distilled water, 2 mL of buffer, 1 unit of Msp I restriction enzyme (Thermo Fisher Scientific, Waltham, MA, USA), and 10 mL of the PCR product. Digestion was carried out at 37°C for 2 h, followed by electrophoresis using a 2% agarose gel in 0.09 M TBE buffer and staining with Safe stain. The resulting gel was then photographed. The size of the DNA fragments was determined by direct comparison. For yeast species other than *Candida albicans*, sequencing was performed to ensure accurate identification. The PCR products were sequenced, and the fasta file sequences were modified, aligned, and submitted to Gene Bank (https://www.ncbi.nlm.nih.gov/genbank/) to obtain an Accession number, after which they were compared with other reference strains from the different sequences. The sequence data were aligned using MEGA 11 (http://www.megasoftware.net/).

### 2.6 Antifungal Susceptibility Test

In vitro antifungal susceptibility patterns assessment of yeast isolates to the following antifungal agents was done according to the recommendations stated in the Clinical and Laboratory Standards Institute (CLSI) M27‐A4 guidelines (Pereira et al. 2021a). The inoculum volumes were prepared by harvesting the cells from 24‐h‐old cultures. The cell densities were adjusted spectrophotometrically in normal saline, resulting in optical densities ranging from 75% to 77% transmission. The final inoculum sizes ranged from 2.5 × 10^3^ to 5 × 10^3^ CFU/mL. The antifungal agents were diluted in RPMI‐1640 medium, which was buffered to pH 7.0 with 0.165 M morpholinepropanesulfonic acid, supplemented with l‐glutamine and without bicarbonate. This resulted in a two‐times concentration of the agents. The diluted agents were then dispensed into a 96‐well microdilution plate, with a final concentration range of 0.064–64 μg/mL for fluconazole, and 0.016–16 μg/mL for itraconazole, clotrimazole, miconazole, ketoconazole, amphotericin B, and flucytosine. Minimum inhibitory concentration (MIC) results for all agents were determined visually after 24–48 h of incubation at 35°C. For amphotericin B, the MIC was defined as the lowest concentration of drug that caused complete growth inhibition. For the other agents, the MIC was defined as the concentration that resulted in a significant (> 50%) decrease in growth. Strains *Candida parapsilosis* (ATCC 22019) and *Candida krusei* (ATCC 6258) were used as quality control measures.

### 2.7 RNA Extraction

Given the importance of inherent resistance in these *N. glabratus* and *P. kudriavzevii*, expression analysis of resistance genes was performed in these species.

The isolates were initially cultured on SDA medium (Merck, Germany) and incubated at 37°C for 24 h. After incubation, the samples were examined using a spectrophotometer, and their optical density (OD) was measured at a wavelength of 600 nm. Each sample was transferred into a sterile microtube (1.5 mL) for RNA extraction. The total RNA extraction kit (Viragene, Iran) was used for this purpose. The NanoDrop Microvolume Spectrophotometer (Thermo Fisher Scientific, USA) was used to qualitatively assess the RNA for all samples. The OD A260/A280 nm ratio of 1.9–2 was observed in all samples, indicating a nucleic acid purity of 90%–100%.

### 2.8 Complementary DNA (cDNA) Synthesis

To ensure the stability of the extracted RNA, cDNA synthesis was conducted immediately using a cDNA synthesis kit (Viragene, Iran) in accordance with the manufacturer's instructions. Specifically, the RNA amounts utilized for cDNA synthesis were standardized within a range of 0.1 ng to 5 μg, and subsequently incorporated into a master mix comprising 2 μL of oligo(dT) and random hexamer primers, along with up to 20 μL of DEPC‐treated water in a sterile microcentrifuge tube. PCR for cDNA synthesis was executed using a temperature cycling protocol that included 10 min at 25°C, 60 min at 47°C, and 5 min at 85°C. Following this process, the samples were stored at −20°C.

### 2.9 Real‐Time Quantitative Reverse Transcription PCR (qRT‐PCR)

The reaction solutions included 1 μL of cDNA, 0.4 μL of each forward and reverse primer for each gene, and 5 μL of Master Mix Green without Rox. Adjusted the final reaction volume to 10 μL by adding 3.2 μL of sterile distilled water. For each sample, qRT‐PCR was performed in triplicate using primers designed based on prior studies (Maheronnaghsh et al. 2022; Chau et al. 2004). Targeting the *ERG11*, *CDR1*, *CDR2*, and *MDR1* genes, in addition to the reference gene *PMA1* (which is widely utilized as an internal control and housekeeping gene in *Candida* spp., Schmitt et al. 2006). The primers employed in this study are presented in Table 1. A negative control, which did not contain a cDNA template, was included for each gene in each assay run.

**Table 1:** Primers used in quantitative reverse transcription polymerase chain reaction.

Genes	Primer sequence (5′ → 3′)	References
*ERG11*	Forward (F) TTGGTGGTGGTAGACATAGATG Reverse (R) AACTATAATCAGGGTCAGGCAC	Maheronnaghsh et al. (2022)
*MDR1*	Forward (F) AGTTGCTTGGGGTAGTTCCG Reverse (R) CTTGCTCTCAACTTTGGTCCG	Maheronnaghsh et al. (2022)
*CDR1*	Forward (F) TGTTGGGTTGGTCTCGATG Reverse (R) TCATAACCTGGACCACTTGG	Maheronnaghsh et al. (2022)
*CDR2*	Forward (F) ATGCCAATGCTGAACCGAC Reverse (R) AAAGTTGTAGCCAAATTAGCAGC	Maheronnaghsh et al. (2022)
*PMA1*	Forward (F) TTGAAGATGACCACCCAATCC Reverse (R) GAAACCTCTGGAAGCAAATTCG	Chau et al. (2004)

Abbreviations: *CDR1*, *Candida* drug resistance1 gene; *CDR2*, *Candida* drug resistance2 gene; *ERG11*, ergosterol 11 (sterol 14‐demethylase) gene; *MDR1*, multidrug resistance1 gene; *PMA1*, plasma membrane ATPase 1 gene.

qRT‐PCR was conducted following a specific thermal cycling protocol: an initial denaturation at 95°C for 15 min (one cycle), followed by 40 cycles of denaturation at 95°C for 15 s and annealing/extension at 60°C for 1 min. Subsequently, a melt curve analysis was performed using the Rotor‐Gene Q (Germany). The expression levels of the genes quantified by qRT‐PCR were expressed in cycle threshold (CT) units. The qRT‐PCR results were analyzed to elucidate the differences in the expression levels of the *ERG11*, *CDR1*, *CDR2*, and *MDR1* genes between fluconazole‐sensitive and resistant groups, as well as clotrimazole‐sensitive and resistant groups. For this analysis, the 2^−∆∆CT^ method was employed. The mean CT values, determined in triplicate for each gene, were utilized for further calculations. To derive the ∆CT value, the mean CT of the *PMA1* reference gene was subtracted from the mean CT values of the target genes *CDR1*, *CDR2*, *ERG11*, and *MDR1*. By comparing the resultant ∆CT values, the fold changes in the expression of the specified genes were determined using the 2^−∆∆CT^ formula.

### 2.10 Statistical Analysis

MIC encompasses the MIC50 (the lowest antifungal concentration that inhibits 50% of growth), the MIC90 (the lowest drug concentration that inhibits 90% of growth), and the geometric mean (GM) was determined. Gene expression data were analyzed using SPSS version 27 software. Inferential statistics and paired sample *t* tests were used to estimate the raw ratio with a 95% confidence interval for different variables. Differences with a *p* < 0.05 were considered statistically significant. To investigate variations in gene expression across different isolates, graphical representations were employed to illustrate and compare the expression levels of individual genes within each isolate group. This approach facilitated a clear and detailed interpretation of the expression patterns for each gene across the various isolates.

## 3 Results

**Figure 1:**
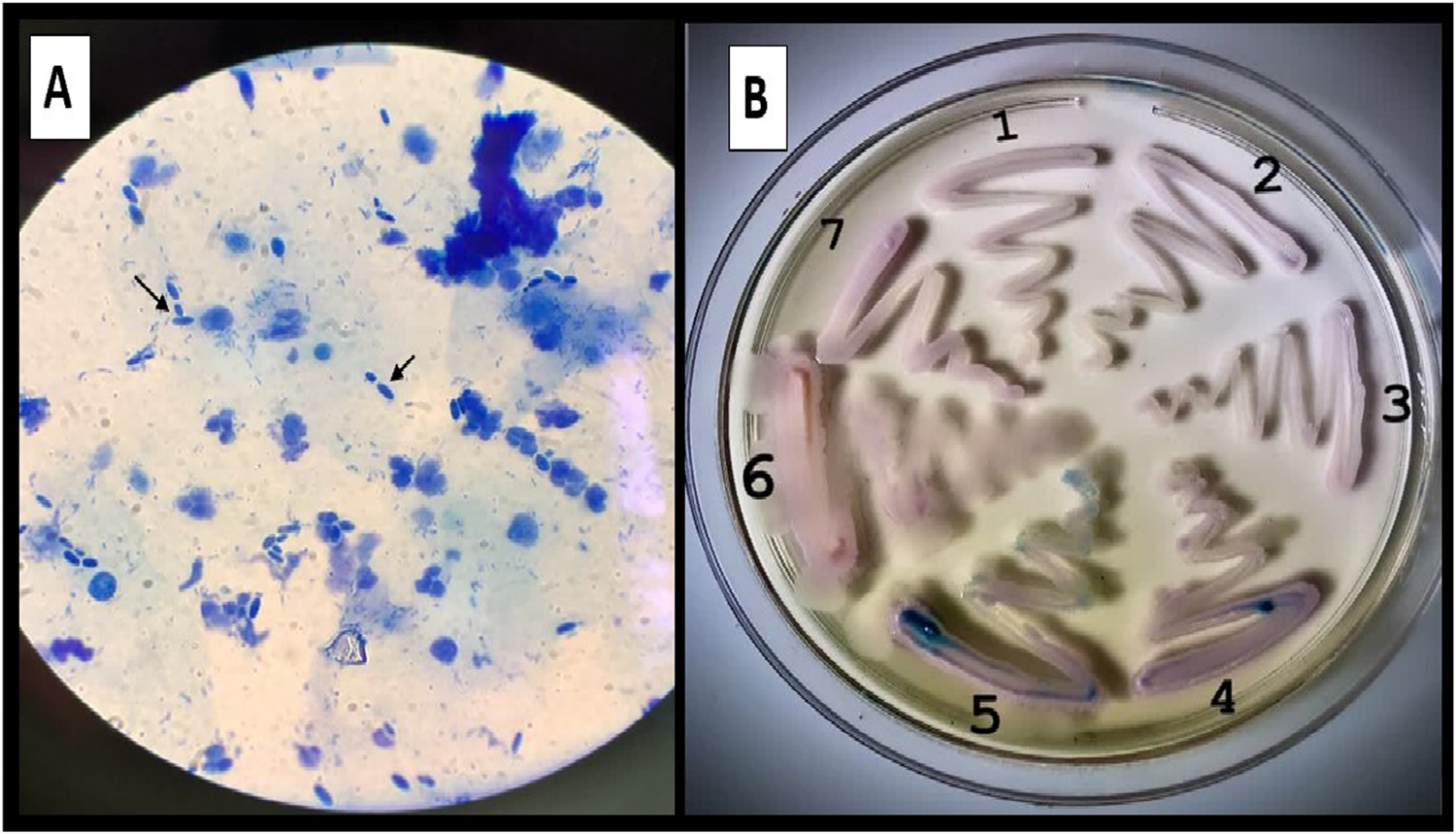
(A) Direct examination of vaginal sample staining with methylene blue and (B) Limited ability of CHROMagar Candida to identify yeast species based on colony colors; 1. *C. parapsilosis*, 2. *S. cerevisiae*, 3. *N. galbratus*, 4 and 5. mix infection with *C. albicans* and *N. glabratus*, 6. *P. kudriavzevii* and 7. *M. guilliermondii*. Colony colors are almost similar, wich increases the possibility of mistakes.

**Figure 2:**
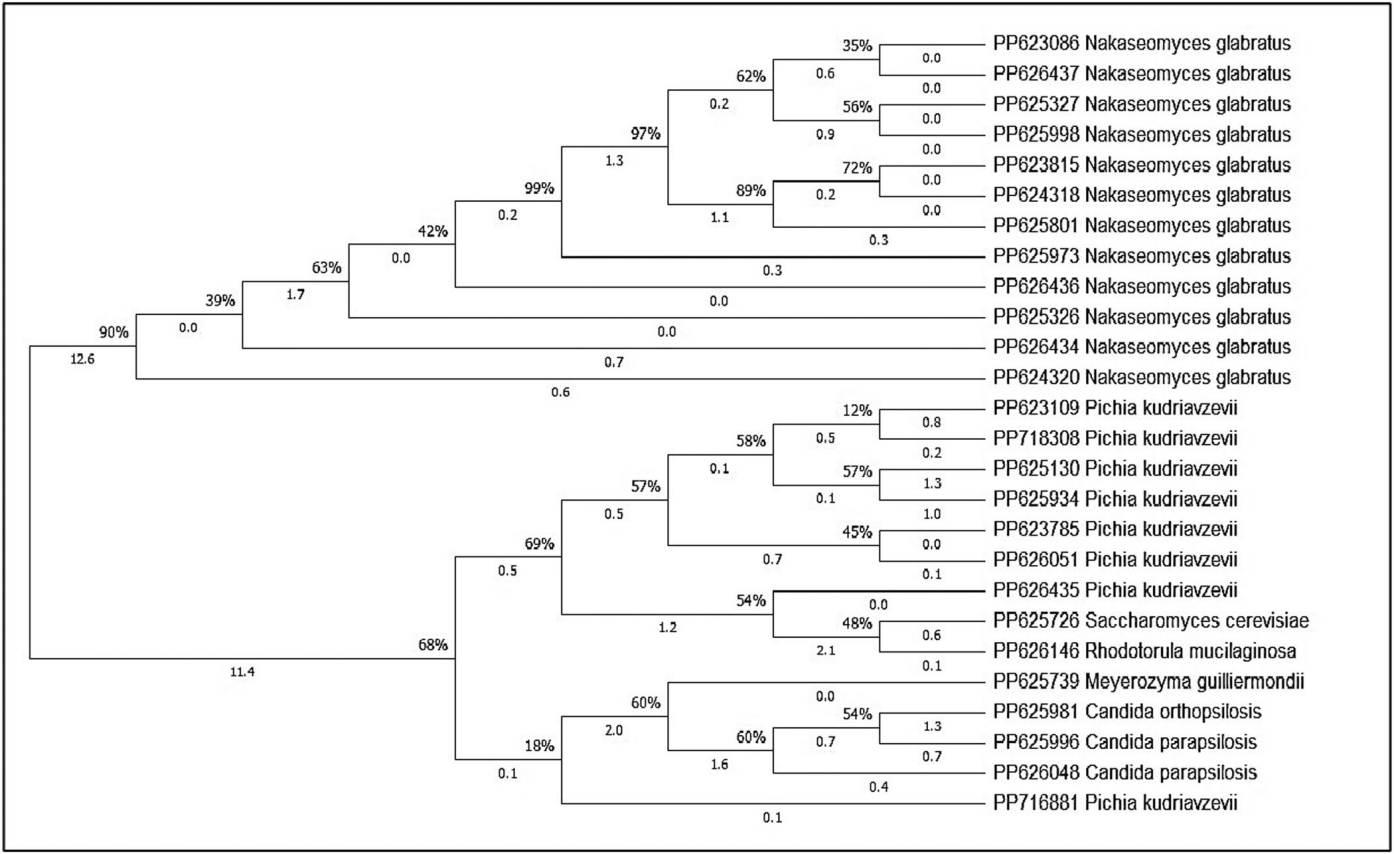
Molecular phylogenetic analysis is using the maximum likelihood method and the Tamura–Nei model with sequences of the ITS region. The tree shows a set of possible nucleotides (states) at each ancestral node based on their inferred likelihood. Initial trees for the heuristic search were obtained automatically by applying Neighbor‐Join and BioNJ algorithms to a matrix of pairwise distances estimated using the Tamura–Nei model, and then selecting the topology with the superior log likelihood value. The rates among sites were treated as being uniform among sites (uniform rates option). This analysis involved 26 nucleotide sequences. There were a total of 794 positions in the final data set. Evolutionary analyses were conducted in MEGA11.

Out of a total of 402 samples suspected of cervicovaginal candidiasis from patients with recurrent episodes that did not respond positively to treatment, 155 samples showed positive yeast cultures, and 45 patients were observed to have cervicovaginal infections caused by yeast species other than *C. albicans*. The average age of the patients was 44.48 years (Figure 1), and the average BMI was 26.95. Among the delivery methods, 21 were cesarean sections, and 20 women had vaginal deliveries, while the remaining cases involved infertility (Table 2). Patients commonly used clotrimazole and metronidazole vaginal ointment without a prescription. Diabetes was present in seven women, while six women had a history of uterine and ovarian problems. Menopause was observed in 15 women (Table 3). A total of 155 samples were analyzed in this study. These samples were carefully selected and included additional yeast species that were identified using various phenotypic tests, including the germ tube test, chlamydoconidia production, color comparison on CHROMagar *Candida* colony medium, and microscopic morphology on corn meal agar. To further confirm the identification, genotypic tests such as restriction fragment length polymorphism (PCR‐RFLP) and ITS‐DNA sequencing were performed (Figure 2). The frequency of yeast species other than *C. albicans* was 29/03% (45/155). The non‐*C. albicans* isolates included *N. glabratus* (*Candida glabrata*) 68% (31/45), followed by *P. kudriavzevii* (*Candida krosei*) (17% (8/45), *C. parapsilosis* 0.04% (2/45), *Meyerozyma guilliermondii* (*Candida guilliermondii*), *Candida orthopsilosis*, *Saccharomyces cerevisiae*, and *Rhodotorula mucilaginosa* with 0.02% (1/45). Additionally, there were 0.04% (2/45) of *P. kudriavzevii*/*C. albicans* and 0.02% (1/45) of *N. glabratus*/*C. albicans* coinfection. The results of the Pap smear sample examination from the patients' cervix revealed the presence of *Candida* spp., and severe inflammation was reported. The drug sensitivity results demonstrated that ketoconazole, itraconazole, and 5‐flucytosine exhibited the highest potency as antifungal agents against *N. glabratus* and *P. kudriavzevii*. Notably, both of these agents displayed a low MIC (MIC_50_ and MIC_90_) and a low GM. These findings indicate that these antifungal agents possess broad effectiveness against these yeast species. However, a subset of *N. glabratus* isolates demonstrated resistance to miconazole, clotrimazole, and amphotericin B. This resistance is discernible through the elevated MIC_50_ and MIC_90_ values, as well as the higher GM for these antifungal agents. Likewise, all isolates of *P. kudriavzevii* exhibited resistance to clotrimazole. *S. cerevisiae* is generally sensitive to all drugs, although it shows more resistance to clotrimazole and miconazole. On the other hand, *C. orthopsilosis* is generally susceptible to all drugs, but specifically resistant to amphotericin B. Similarly, *C. parapsilosis* is generally susceptible to all drugs, but isolates show increasing resistance to amphotericin B. In the case of *M. guilliermondii*, the highest resistance was related to amphotericin B. *R. mucilaginosa* showed the highest resistance to fluconazole (Table 4).

**Table 2:** The distribution of yeast species among a total of 45 patients, categorized according to their clinical symptoms and cervicovaginal conditions.

	Symptoms				Cervicovaginal condition		
Yeast organism(s) isolated	Dyspareunia	Discharge	Itching	Irritation	Cheesy discharge	Discharge mixed with inflammation	Ectropion and ulcers
*Nakaseomyces glabratus* (*N* = 31)	21	31	20	10	14	15	2
*Pichia kudriavzevii* (*N* = 8)	5	8	6	5	0	6	2
*Candida parapsilosis* (*N* = 2)	1	2	1	0	0	2	0
*Candida orthopsilosis* (*N* = 1)	0	1	0	0	0	1	0
*Meyerozyma guilliermondii* (*N* = 1)	0	1	1	1	0	1	0
*Saccharomyces cerevisiae* (*N* = 1)	0	1	0	1	0	1	0
*Rhodotorula mucilaginosa* (*N* = 1)	0	1	0	0	1	0	0
Sum	27	45	28	17	15	26	4

**Table 3:** Yeast species distribution among 45 patients with recurrent cervical infections based on their demographic information.

	BMI				Method of delivery			Period conditions			Diabete	Laser removal of genital hair history
Species	16–18/5	18/5–24	24–30	≥ 30	NVD	S/C	Inf	Reg	Irreg	Meno		
*Nakaseomyces glabratus* (*N* = 31)	2	8	14	7	14	14	3	14	7	10	6	5
*Pichia kudriavzevii* (*N* = 8)	0	1	5	2	2	6	0	5	0	3	1	3
*Candida parapsilosis* (*N* = 2)	0	0	2	0	2	0	0	0	1	1	0	0
*Candida orthopsilosis* (*N* = 1)	0	0	1	0	0	0	1	1	0	0	0	0
*Meyerozyma guilliermondii* (*N* = 1)	0	0	1	0	0	1	0	0	1	0	0	0
*Saccharomyces cerevisiae* (*N* = 1)	0	0	1	0	1	0	0	0	0	1	0	0
*Rhodotorula mucilaginosa* (*N* = 1)	0	0	1	0	1	0	0	0	1	0	0	1
Sum	2	9	25	9	20	21	4	20	10	15	7	9

Abbreviations: BMI, body mass index; Inf, infertility; Irreg, irregular; Meno, menopause; NVD, natural vaginal delivery; Reg, regular; S/C, cesarean section.

**Table 4:** In vitro antifungal susceptibility of seven antifungal agents against 45 yeast species isolated from patients with recurrent cervicovaginal infection.

Species		FLU (µg/mL)	MICO (µg/mL)	CLO (µg/mL)	KET (µg/mL)	ITR (µg/mL)	5‐FC (µg/mL)	AMB (µg/mL)
*Nakaseomyces glabratus* (*N* = 31)	MIC 50	2	16	16	1	1	1	16
	MIC 90	8	16	16	2	2	1	16
	GM	2.74	10.07	10.74	1.51	1.24	1	15.48
	MIC Range	0.0625–64	1–16	0.0312–16	0.0156–16	0.0312–2	0.0312–1	0.0156–16
*Pichia kudriavzevii* (*N* = 8)	MIC 50	8	16	16	16	2	1	16
	MIC 90	36	16	16	16	3.4	1.8	16
	GM	8.97	10.76	16	8	2	1.25	8.97
	MIC Range	0.0625–64	1–16	0.25–16	1–16	0.125–4	0.25–2	0.0156–16
*Saccharomyces cerevisiae*	MIC	2	16	16	0.0156	0.5	0.5	0.0156
*Candida orthopsilosis*		0.25	0.25	0.25	0.0156	0.125	0.25	16
*Candida parapsilosis* ^*isolate 1*^		0.5	1	0.5	0.25	1	0.5	16
*C. parapsilosis* ^*isolate 2*^		0.5	0.25	0.0625	0.0312	1	0.5	0.0156
*Meyerozyma guilliermondii*		1	1	0.5	0.0312	1	1	16
*Rhodotorula mucilaginosa*		64	2	2	0.0312	2	0.5	0.0312

Abbreviations: 5‐FC, 5‐fluorocytosine; AMB, amphotericin B; CLO, clotrimazole; FLU, fluconazole; GM, geometric mean; ITR, itraconazole; KET, ketoconazole; MIC, minimum inhibitory concentration; MICO, miconazole.

The initial stage of patient culture yielded over 1000 colonies per plate. In the subsequent stage involving 11 patients, it was determined that the patients were infected with *P. kudriavzevii* and *N. glabratus*. In the third stage of culture, however, only one colony was observed in these patients.

The 11 patients in the first stage were classified as CLR isolates due to their resistance to treatment in both clinical and laboratory settings. The second stage isolates, which exhibited resistance only in the clinical context while being reported as drug‐susceptible in laboratory analysis, were designated as CR isolates. The third stage isolates, demonstrating sensitivity in both clinical and laboratory environments, were classified as clinical and laboratory sensitive (CLS) isolates. Subsequently, the samples were analyzed to assess the expression of resistance genes at each stage of the process.

For the analysis of gene expression data, each group of isolates was compared against the others and presented in a tabular format for comparative analysis (Table 5).

**Table 5:** Expression of resistance genes in *Nakaseomyces glabratus* and *Pichia kudriavzevii* groups.

	*ERG11*	*CDR1*	*CDR2*	*MDR1*
***N. glabratus***				
CR and CLS average ratio ± Standard error	0/53 ± 0/87	21/53 ± 1/26	1/84 ± 1/31	1/59 ± 1/12
(*t* test) *p* value	0/60	0/28	0/64	0/65
CLR and CLS average ratio ± Standard error	1/77 ± 51/25	0/67 ± 84/96	1/66 ± 6/92	0/52 ± 5/51
(*t* test) *p* value	0/24	0/12	0/36	0/06
***P. kudriavzevii***				
CR and CLS average ratio ± Standard error	2/16 ± 28/56	1/33 ± 14/83	0/02 ± 4/84	0/21 ± 15/73
(*t* test) *p* value	0/23	0/25	0/04	0/004
CLR and CLS average ratio ± Standard error	1/22 ± 3/08	0/35 ± 35/89	0/64 ± 2/19	0/86 ± 6/10
(*t* test) *p* value	0/13	0/04	0/37	0/25

Abbreviations: *CDR1*, *Candida* drug resistance1 gene; *CDR2*, *Candida* drug resistance2 gene; CLR, clinical and laboratory resistant; CLS, clinical and laboratory sensitive; CR, clinical resistant; *ERG11*, ergosterol 11 (sterol 14‐demethylase) gene; *MDR1*, multidrug resistance1 gene.

### 3.1 ERG11

**Figure 3:**
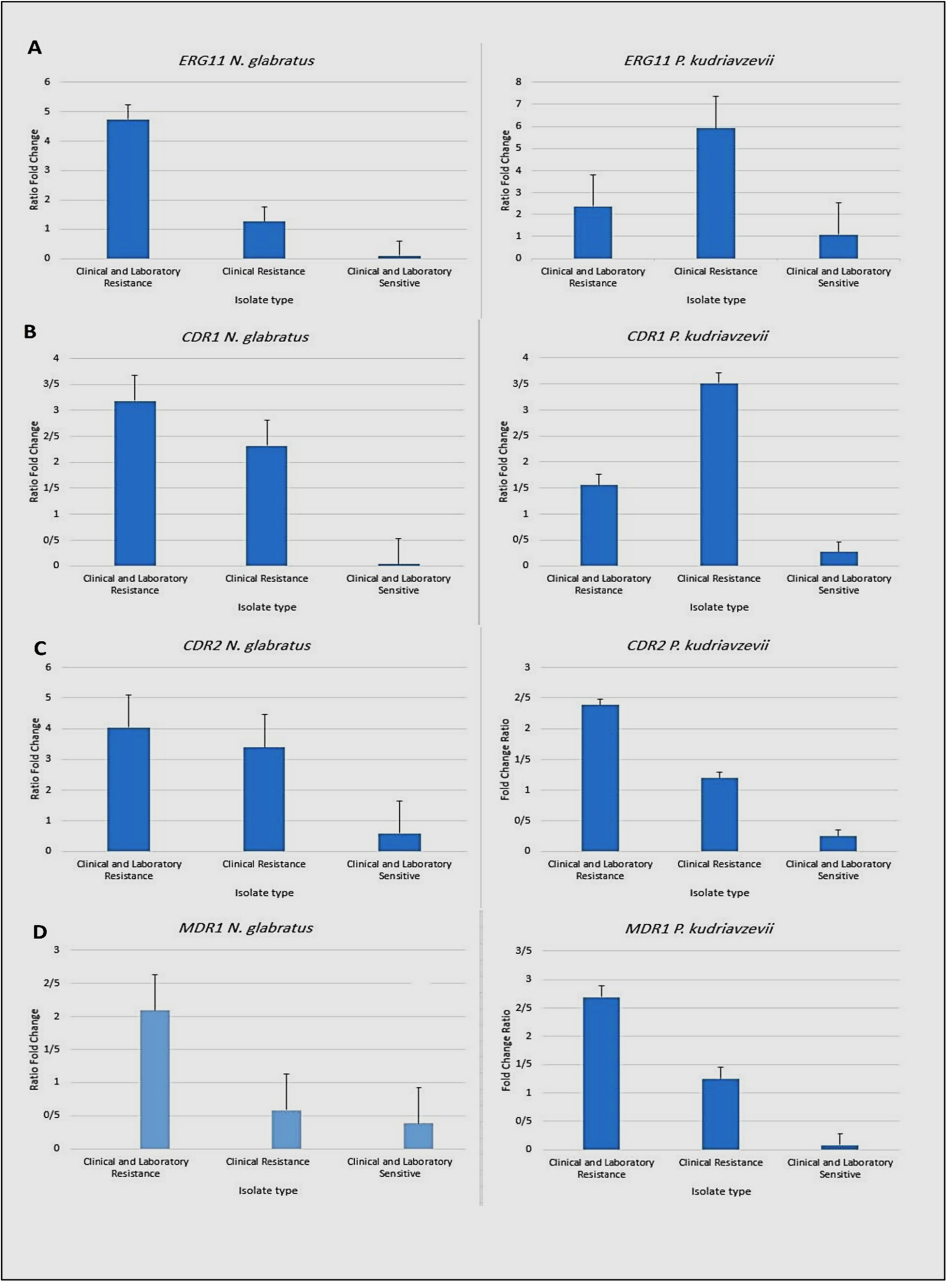
(A) Comparison of *ERG11* gene expression in *Nakaseomyces glabratus* and *Pichia kudriavzevii* in three groups, (B) comparison of *CDR1* gene expression in *N. glabratus* and *P. kudriavzevii* in three groups, (C) comparison of *CDR2* gene expression in *N. glabratus* and *P. kudriavzevii* in three groups, and (D) Comparison of *MDR1* gene expression in *N. glabratus* and *P. kudriavzevii* in three groups. *CDR1*, *Candida* drug resistance1 gene; *CDR2*, *Candida* drug resistance2 gene; *ERG11*, ergosterol 11 (sterol 14‐demethylase) gene; *MDR1*, multidrug resistance1 gene.

In *N. glabratus*, the CLR group exhibits the highest rate of change in gene expression. In contrast, the CR group demonstrates a comparatively lower level of expression change. Meanwhile, the CLS group exhibits minimal changes in gene expression. In *P. kudriavzevii*, the CR group exhibits the highest rate of change in gene expression. In contrast, the CLR group demonstrates a comparatively lower level of expression change. The CLS group showed greater expression compared with the CLS group of *N. glabratus* isolates (Figure 3A).

### 3.2 CDR1

The CLR group in *N. glabratus* exhibits the highest rate of change in gene expression, and the CR group demonstrates a lower level of expression change. But in *P. kudriavzevii*, the CR group exhibits the highest rate of change in gene expression. In contrast, the CLR group demonstrates a comparatively lower level of expression change. The CLS group showed more expression compared with the CLS group of *N. glabratus* isolates (Figure 3B).

### 3.3 CDR2

In the *CDR2* gene, a comparable expression pattern was observed in both species, with the highest expression levels noted in the CLR group. Furthermore, *P. kudriavzevii* exhibited greater expression of this gene compared with *N. glabratus* (Figure 3C).

### 3.4 MDR1

Although the gene expression pattern was similar for the *MDR1* gene, differences in gene expression levels were observed between species. In *P. kudriavzevii*, the CR group exhibited higher expression levels compared with *N. glabratus*. Additionally, within the CLS isolates group, there was a greater increase in expression compared with *P. kudriavzevii* (Figure 3D).

## 4 Discussion

In recent years, there has been a notable shift in the epidemiology of vulvovaginal candidiasis (VVC). Cervicovaginal candidiasis possesses the capacity to impact a wide range of patients, including those who exhibit symptoms and those who demonstrate positive culture outcomes without any visible symptoms (Adad et al. 2001). This condition can manifest with various symptoms, ranging from asymptomatic instances to severe and incapacitating presentations (Brandolt et al. 2017). The severity and presentation of symptoms in RCVC can vary widely among individuals. Factors that may influence symptom severity in patients include immune system status, hormonal fluctuations, and the presence of other underlying health conditions, such as diabetes (Wasim et al. 2021; Bahat et al. 2024). In this study, repeated laser sessions for genital hair removal were documented in the medical history of 20% (9/45) of patients. Although the etiology of this factor remains poorly understood, it is possible to hypothesize that it plays a role in the pathogenesis of the infection (Graziottin 2024; Klann et al. 2019). Further research is needed to determine whether this issue can be identified as a risk factor. The stages of infection involve colonization, superficial penetration of epithelial cells, and the spread of infection. The epithelial cells of the cervix and vagine not only encounter mechanical and barrier challenges through mucin and keratin, but they also possess the capability to recognize danger signals from pathogens and initiate immune responses (Pandey et al. 2021). Studies did not observe a clear distinction between the vaginal microbiota of females with a history of RCVC and healthy controls. This suggests that the vaginal microbiota does not confer protection against any form of infection (Godoy‐Vitorino et al. 2018). The precise factors that contribute to the transition from asymmetrical cervicovaginal infection to RCVC are still undefined (Cengiz et al. 2020). Although some recurrences can be attributed to predisposing factors associated with CVC itself, many cases of RCVC occur in women without any identifiable risk factors (Brandolt et al. 2017). According to a study conducted by Moradi et al., a significant fungus identified as *C. albicans* was found to be the connecting factor between cervicovaginal infections and cervical cancer (Moradi et al. 2017). A number of studies have documented a rising prevalence of non‐*C. albicans* isolates in instances of vaginitis and infections attributed to atypical yeasts. The diagnosis of cervicovaginal fungal infection is frequently made based on the identification of characteristic symptoms (Cengiz et al. 2020). May result in imprecision due to its limited specificity. Identifying clinically significant *Candida* strains is crucial for improving the effectiveness of treatment and ensuring timely eradication (Donders et al. 2022; Borman and Johnson 2023). While *C. albicans* remains the primary causative agent, infections caused by NAC species, such as *N. glabratus* and *P. kudriavzevii*, have increased significantly (Makanjuola et al. 2018). In this study, *N. glabratus* was found to be the second most common yeast, after *C. albicans*, contrary to the findings of Jannati et al. (2024). This epidemiological shift is largely attributed to the growing resistance of NAC species to antifungal medications (Jamiu et al. 2020). The study by Behzadi et al. (2015) demonstrates that the distribution of prevalent NAC species in urinary tract infections, including *N. glabratus* and *C. tropicalis*, varies regionally across different parts of the world, including Brazil, the United States, India, and Iran. Furthermore, these species are implicated in the etiology of reccurent vulvovaginal candidiasis infections in women. Additional research indicates a significant increase in VVC attributable to *N. glabratus* among older women, particularly in the Iranian population Behzadi et al. 2015.

While relatively infrequent, multiple studies have provided evidence pointing to *S. cerevisiae* as the predominant responsible agent for genitourinary infections. In this study, similar to the research conducted by Gaziano et al., a single instance of *S. cerevisiae* was detected in the genital region (Gaziano et al. 2020). *M. guilliermondii* is an infrequent yeast pathogen associated with the severe condition of invasive candidiasis. Moreover, it is one of the less prevalent strains that have been recognized as a causative agent of non‐*C. albicans* vaginitis (Jannati et al. 2024). In addition, a yeast species called *R. mucilaginosa* was also isolated. Recent research has shown that it can also act opportunistically. In this study, it was found in one sample, similar to Pereira et al. Although the prevalence of this species in studies was relatively low, its presence can confirm the occurrence of vaginal infection caused by *R. mucilaginosa* in symptomatic individuals (Pereira et al. 2021). Treatment failure is a prevalent issue in cases of non‐*C. albicans* species. This can be attributed to the inherent resistance or limited susceptibility of certain species within this group to commonly employed antifungal medications (Shi et al. 2020a). DNA‐based methodologies offer significant advantages for carrying out thorough epidemiological investigations on non‐*C. albicans* species (Codreanu and Ciurea 2023). Molecular methodologies have the potential to improve the discrimination of less prevalent yeast isolates and closely related yeast species, specifically those belonging to the *Candida* complexes (Shi et al. 2020a). In vitro studies have demonstrated varying degrees of resistance among *Candida* species. In our investigation, the resistance levels to fluconazole and miconazole were found to be higher compared with the study conducted by Brandolt et al (Brandolt et al. 2017). Nevertheless, a separate study conducted in Santa Catarina by Dalazen et al. revealed a 100% resistance rate to fluconazole (Dalazen et al. 2011). As observed in previous studies, *N. glabratus* exhibited significant resistance to azole drugs (Yassin et al. 2020; Lotfali et al. 2022).

Multidrug resistance (*MDR*) genes encode proteins known as *MDR* efflux pumps. These pumps are located in the fungal cell membrane and actively remove a wide range of drugs, including azoles, from the cell. This action reduces the intracellular concentration of the drug, thereby diminishing its effect. MDR pumps transport the drug from inside the cell to the outside using energy derived from ATP. These pumps are capable of transporting a diverse array of drugs, which contributes to MDR in fungi. Increased expression of *MDR* genes is one of the primary mechanisms of azole resistance in *N. glabratus* and *P. kudriavzevii*. This increased expression can occur due to mutations in the regulatory regions of the genes or due to heightened expression of transcription factors (Catalano et al. 2022; Nishino et al. 2021).

*CDR* Genes encode proteins known as *CDR* efflux pumps. These pumps, located in the fungal cell membrane, actively remove antifungal drugs, including azoles, from the cell. *CDR* pumps exhibit more specificity than MDR pumps. They also transport drugs from the inside of the cell to the outside using energy from ATP. Certain CDR pumps, such as *CDR1* and *CDR2* in *C. albicans*, are specifically involved in the transport of fluconazole. Increased expression of *CDR* genes is also a significant mechanism of azole resistance in *N. glabratus* and *P. kudriavzevii*. In *N. glabratus*, heightened expression of these genes plays a crucial role in fluconazole resistance (Holmes et al. 2016).

The *ERG11* gene encodes the enzyme 14‐α‐lanosterol demethylase, which is involved in the synthesis of ergosterol, a major component of the fungal cell membrane (Flowers et al. 2015). Azoles function by inhibiting this enzyme. Mutations in the *ERG11* gene can lead to alterations in the structure of the enzyme 14‐α‐lanosterol demethylase. These changes can reduce the affinity of azoles for binding to the enzyme, rendering the drug ineffective in inhibiting its function. Consequently, ergosterol synthesis continues, and the fungal cell membrane maintains its normal function (Xiang et al. 2013). Alterations in the *ERG11* gene are among the primary mechanisms of azole resistance in *N. glabratus* and *P. kudriavzevii*. Various mutations have been identified in this gene that can confer resistance to different azoles (Hossain et al. 2022).

A notable contrast is observed when comparing the gene expression patterns between the two species Liu et al. 2021. In *N. glabratus*, there is a consistent trend towards higher expression of all investigated genes in the CLR group, suggesting a potential synergistic effect of multiple resistance mechanisms in this group Shi et al. 2020b; Abd‐Alrahman et al. 2021. This is particularly evident for *CDR1* and *CDR2*, which are efflux pumps known to confer resistance to a wide range of antifungal agents Esfahani et al, 2024; Maheronnaghsh et al. 2022. The increased expression of *ERG11*, which encodes the lanosterol 14‐α‐demethylase enzyme crucial for ergosterol biosynthesis, may indicate compensatory mechanisms to counteract the effects of azole drugs (Sanchez Villacreses 2025; Liu et al. 2021). In *N. glabratus*, the expression of *ERG11* was found to be increased when compared with the susceptible group, which aligns with the findings reportedby Lotfali et al. (2022) (Lotfali et al. 2022). While *N. glabratus* showed increased *ERG11* expression in the CLR group, *P. kudriavzevii* exhibited higher *ERG11* expression in the CR group. These findings suggest that the role of *ERG11* in azole resistance may be more complex than previously thought and could vary between species (Abd‐Alrahman et al. 2021; Esfahani et al. 2022). In contrast, in *P. kudriavazevii*, a more complex scenario unfolds. While *CDR1* expression is elevated in the CLR group, the expression of *ERG11* and *MDR1* is paradoxically higher in the CR group. The levels of *CDR1* and *ERG11* overexpression in *N. glabratus* showed similar results to the study conducted by Shi et al. (2020b) when compared with other genes. Interestingly, in *P. kudriavzevii*, the expression of *CDR1* was found to be higher in the CLR group, whereas the CR group exhibited higher expression of *CDR2*. This discrepancy warrants further exploration to elucidate the underlying mechanisms. It is conceivable that different resistance pathways predominate in the two resistance phenotypes, or that the interplay between these genes is more intricate in *P. kudriavzevii*.

Implications for antifungal resistance, the observed differences in gene expression profiles between the two species highlight the diverse strategies employed by fungi to develop resistance (Maheronnaghsh et al. 2022; Esfahani et al. 2022). The upregulation of multiple resistance genes in *N. glabratus* underscores the potential for rapid acquisition of MDR in this species, emphasizing the need for judicious antifungal use (Makanjuola et al. 2018; Liu et al. 2021; Zaman et al. 2022; Shokoohi et al. 2020). In contrast, the more complex resistance landscape in *P. kudriavzevii* suggests that targeting single resistance mechanisms might be insufficient and that combination therapies or novel antifungal agents may be required. While the specific focus on *N. glabratus* and *P. kudriavzevii* is novel, the overall trends in gene expression are consistent with previous reports on other fungal species (Maheronnaghsh et al. 2022). For instance, the upregulation of *ERG11* and efflux pump genes in response to azole exposure has been well‐documented. However, the relative contribution of different resistance mechanisms to the overall phenotype can vary depending on the fungal species, the specific antifungal agent, and the clinical setting (Makanjuola et al. 2018). Furthermore, the absence of statistically significant disparities in some gene expression in *N. glabratus* and *P. kudriavzevii* implies that additional factors, such as genetic mutations or epigenetic modifications, might play a role in the development of resistance in these species.

Our findings suggest that in certain strains that show intrinsic resistance to antifungal agents such as fluconazole and clotrimazole, gene expression may decrease during treatment, as was observed in *N. glabratus*. Conversely, in other strains, such as *P. kudriavzevii*, gene expression may increase during treatment. This observed pattern may indicate the possibility of frequent relapse and the emergence of a chronic form of infection among patients in the absence of long‐term follow‐up and complete treatment, although this requires further investigation, and this study will serve as a basis for future studies.

Future directions and further investigations are needed to elucidate the precise roles of the studied genes in the development of antifungal resistance in *N. glabratus* and *P. kudriavzevii*. Functional studies, such as gene deletion or overexpression experiments, could provide valuable insights into the contribution of individual genes to the resistant phenotype. Additionally, exploring the interactions between different resistance mechanisms and their impact on antifungal susceptibility would enhance our understanding of the complex nature of fungal resistance.

## 5 Conclusions

These results indicate that elevated gene expression may persist even when an isolate is classified as susceptible based on drug susceptibility testing. Therefore, treatment should not be discontinued solely based on the laboratory‐determined susceptibility status of the isolate. These findings underscore the need to investigate drug resistance in real clinical settings, as relying exclusively on laboratory results may not yield a complete understanding of resistance mechanisms.

### 5.1 Limitation

The limitation of this study is that it focused exclusively on *N. glabratus* and *P. kudriavzevii*, which may not adequately represent the entire spectrum of NAC species. However, due to their characteristic resistance to azoles, these two species are more prevalent compared with others. Additionally, this study primarily concentrated on a few known resistance mechanisms (*ERG11*, *CDR1*, *CDR2*, and *MDR1*) that appear to play more significant roles, while other mechanisms, such as mutations in target genes and alterations in ergosterol biosynthesis, are also important.

## Author Contributions

**Fatemeh Zahra Ranjbar Golafshani:** conceptualization, investigation, writing original draft, methodology, validation, data curation, software. **Soheila Abbaszadeh Godarzi:** conceptualization, investigation, writing original draft, methodology, validation, data curation. **Firoozeh Kermani:** software, formal analysis, investigation, writing – review and editing, visualization. **Saeid Mahdavi Omran:** visualization, validation, resources, writing – review and editing, project administration, supervision.

## Ethics Statement

Written informed consent was obtained from the patients before the commencement of this study. The study was conducted by Babol University of Medical Sciences in compliance with the ethical guidelines specified under code IR.MUBABOL.HRI.REC.1402.138. Consent to participate informed consent was obtained from all individual participants included in the study.

## Consent

The authors affirm that human research participants provided informed consent for publication.

## Conflicts of Interest

The authors declare no conflicts of interest.

## Data Availability

The data that support the findings of this study are available on request from the corresponding author. The data are not publicly available due to privacy or ethical restrictions.
